# Adoptive cell therapy using PD-1^+^ myeloma-reactive T cells eliminates established myeloma in mice

**DOI:** 10.1186/s40425-017-0256-z

**Published:** 2017-06-20

**Authors:** Weiqing Jing, Jill A. Gershan, Grace C. Blitzer, Katie Palen, James Weber, Laura McOlash, Matthew Riese, Bryon D. Johnson

**Affiliations:** 1 Division of Hematology/Oncology/Transplant, Department of Pediatrics, Medical College of Wisconsin, Milwaukee, WI 53226 USA; 2 Medical Student, Medical College of Wisconsin, Milwaukee, WI 53226 USA; 3 Division of Hematology/Oncology, Department of Medicine, Medical College of Wisconsin, Milwaukee, WI 53226 USA

**Keywords:** Myeloma, Adoptive cell therapy, PD-1, PD-L1, Cancer-infiltrating lymphocytes

## Abstract

**Background:**

Adoptive cellular therapy (ACT) with cancer antigen-reactive T cells following lymphodepletive pre-conditioning has emerged as a potentially curative therapy for patients with advanced cancers. However, identification and enrichment of appropriate T cell subsets for cancer eradication remains a major challenge for hematologic cancers.

**Methods:**

PD-1^+^ and PD-1^−^ T cell subsets from myeloma-bearing mice were sorted and analyzed for myeloma reactivity in vitro. In addition, the T cells were activated and expanded in culture and given to syngeneic myeloma-bearing mice as ACT.

**Results:**

Myeloma-reactive T cells were enriched in the PD-1^+^ cell subset. Similar results were also observed in a mouse AML model. PD-1^+^ T cells from myeloma-bearing mice were found to be functional, they could be activated and expanded ex vivo, and they maintained their anti-myeloma reactivity after expansion. Adoptive transfer of ex vivo-expanded PD-1^+^ T cells together with a PD-L1 blocking antibody eliminated established myeloma in Rag-deficient mice. Both CD8 and CD4 T cell subsets were important for eradicating myeloma. Adoptively transferred PD-1^+^ T cells persisted in recipient mice and were able to mount an adaptive memory immune response.

**Conclusions:**

These results demonstrate that PD-1 is a biomarker for functional myeloma-specific T cells, and that activated and expanded PD-1^+^ T cells can be effective as ACT for myeloma. Furthermore, this strategy could be useful for treating other hematologic cancers.

## Background

Multiple myeloma (MM) is an incurable hematologic malignancy characterized by the clonal expansion of malignant plasma cells. Despite aggressive therapy including chemotherapy and hematopoietic stem cell transplantation (HSCT), most patients die from disease relapse. Immunotherapies including adoptive T cell therapy and checkpoint inhibitors have been used to treat a variety of solid and hematologic cancers with remarkable clinical responses in a subset of patients [1–4]. However, identifying which immunotherapy or combination thereof is effective for rejection of multiple myeloma remains a challenge.

In the past decade, our laboratory has been exploring immunotherapy approaches for the treatment of MM using the MHC class I-expressing murine 5T33 myeloma model. In our initial studies, we showed a unique combination of therapy was able to halt 5T33 disease progression in mice. A combinatorial approach consisting of lethal whole body irradiation (WBI), bone marrow transplantation (BMT) and adoptive T cell transfer, plus treatment with a cancer vaccine and anti-PD-L1, resulted in a 100-day survival rate of 40% for 5T33-bearing mice [5]. This compared to 0% survival of mice treated with vaccine alone, anti-PD-L1 alone, or vaccine and anti-PD-L1 without WBI, BMT and naïve T cell transfer. When anti-PD-L1 therapy was combined with lethal WBI, BMT, and transfer of myeloma antigen-experienced T cells (i.e., from 5T33-bearing donor mice instead of naïve mice), 100% of myeloma-inoculated mice survived to day 100 [6]. Together, these studies highlighted critical components required to induce anti-cancer immunity against 5T33 myeloma. Activation of myeloma antigen-specific lymphocytes or adoptive transfer of cancer antigen experienced T cells in a lymphopenic setting, followed by checkpoint blockade, appear to be required to activate and maintain 5T33-specific T cells. Notably, in follow-up studies, the immunotherapy platform was simplified to include non-myeloablative WBI (400–500 cGy) followed by anti-PD-L1 therapy. This combination provided protection from myeloma disease progression in 40% of mice over 100 days [6]. Since there was no transfer of T cells, it appeared that radiation-resistant, myeloma-specific T cells were activated under the conditions of lymphopenia and immune checkpoint blockade. Anti-PD-L1 therapy without non-myeloablative WBI was ineffective.

These earlier studies provided critical insights into myeloma immunity. The murine 5T33 myeloma expresses PD-L1, and the malignant cells reside in the bone marrow and spleen, with few myeloma cells detectable in the blood or other tissues. T cells expressing PD-1 are not detected in the blood, but are detected in the bone marrow and spleen. As the myeloma burden progresses, percentages of PD-1^+^CD4^+^ and CD8^+^ T cells correspondingly increase [5]. While it has been known for several years that PD-1 expression is an indicator of T cell dysfunction under conditions of chronic antigen stimulation [7, 8], more recently it was documented that cancer antigen-reactive T cells in solid tumors express PD-1 [9]. In melanoma tumors, PD-1 was shown to be a marker of functional cancer antigen-reactive tumor-infiltrating T lymphocytes (TIL) [10–12]. Based on these results, we hypothesized that immune therapy for the treatment of myeloma could be further improved by infusion of myeloma antigen-specific PD-1^+^ T cells in the context of lymphopenia and immune checkpoint blockade. The goal of the current study was to enrich for PD-1^+^ myeloma antigen-specific T cells and demonstrate their anti-myeloma efficacy in vivo. Since cancer antigens for myeloma (as well as many other cancers) are unknown, this polyclonal approach for T cell ACT is desirable. It would target cancer cells with heterogeneous mutational landscapes. Furthermore, regarding clinical translation, this process would avoid the technical challenges required to genetically modify T cells to express specific cancer antigen receptors (eg., chimeric antigen receptors or TCRs).

In this study, we isolated and characterized the 5T33-antigen experienced PD-1^+^ T cells, and used them as adoptive T cell therapy (ACT) in combination with a PD-L1 blocking antibody in Rag1-deficient mice. Rag1-deficient mice were used as immunotherapy recipients as they provided a ‘clean’ system to assess the anti-myeloma effects provided by adoptively transferred T cells. More specifically, Rag-1 mice are constitutively lymphopenic (i.e., there is no need for WBI), and there are no endogenous T cells that would be impacted by PD-1/PD-L1 blockade. The presence of endogenous T cells would have made it difficult to clearly assess the anti-myeloma effects of the infused T cells. We found that myeloma antigen-experienced PD-1^+^ T cells can be activated ex vivo to proliferate. They produced IFN-γ, similar to PD-1^−^ T cells, but had a unique cytokine profile producing both IFN-γ and IL-10. As in the solid cancer melanoma [9, 13, 14], the 5T33 myeloma-reactive T cells were found to reside in the PD-1^+^ cell subset. Notably, when PD-1^+^ T cells were given as ACT in vivo plus PD-L1 blocking antibody, a robust anti-5T33 immune response was induced. Thus, in this hematologic malignancy model, it is clear that PD-1^+^ T cells can be activated to expanded ex vivo, produce Th1 cytokines, and provide an anti-myeloma effect in vivo. To the best of our knowledge, this is the first study to use PD-1^+^ T cells in vivo for ACT in hematologic malignancies.

## Methods

### Mice

C57BL/KaLwRij (KaLwRij), (KaLwRij × C57BL/6.SJL)F_1_ and Rag-1-deficient C57BL/6 mice were bred and housed in the Medical College of Wisconsin (MCW) Biomedical Resource Center. C57BL/6 mice were purchased from The Jackson Laboratory (Bar Harbor, ME).

### Myeloma model

The 5T33 murine myeloma cell line was derived from a spontaneous myeloma that arose in a C57BL/KaLwRij mouse. 5T33 cells were engineered to express emerald green fluorescent protein (5T33-GFP), as previously described [6]. CD80 expressing 5T33 (5T33-CD80) were derived by transducing 5T33 cells with a lentiviral expression vector (PLVX-N1; Clontech, Mountain View, CA) encoding the CD80 gene. The 5T33 cell line was transduced with a lentivector to express the ovalbumin (OVA) model antigen MHC class I (SIINFEKL; aa257–264). The vector pLVX-mCherry-N1 (Clontech #632562) was modified by replacing the mCherry gene sequence with a synthetic gene fragment containing OVA peptide sequences (custom gBlock gene fragment from IDT). A 5T33 cell clone stably expressing the OVA peptide was selected by limiting dilution.

Mice were inoculated with 2 × 10^6^ 5T33, 5T33-GFP, 5T33-GFP-OVA or C1498.SIY cells intravenously (iv). Myeloma-bearing mice were considered moribund when they developed paraparesis or paraplegia and were euthanized. C1498-SIY murine AML cells were kindly provided by Dr. Justin Kline at the University of Chicago.

### PD-1^+^ T cell sorting and ex vivo expansion

Sorting of PD-1^+^ or PD-1^−^ T cells from 5T33-myeloma bearing mice was performed using a FACSAria flow cytometric sorter. T cells were activated and expanded in culture with plate-bound anti-CD3 mAb (clone 145-2C11, BD Biosciences; 5 μg/mL) and anti-CD28 mAb (clone 37.51, BD Biosciences; 1 μg/mL) in the presence of IL-2 (20 U/ml), IL-7 (5 ng/ml) and IL-15 (5 ng/ml) for 7 days.

### ACT experiments

Rag-1-deficient mice were injected iv with 1 × 10^6^ 5T33 cells. Five days after myeloma inoculation, the mice received ACT consisting of 3–4 million expanded T cells (1:1, CD8^+^:CD4^+^ ratio) or 2 million expanded CD8^+^ or CD4^+^ T cells injected iv. Treatment with anti-PD-L1 (125 μg intraperitoneally) was given on days 5, 8, 12 and 17 or days 7, 10, 14 and 17 after 5T33 inoculation depending on the experiment. Myeloma-bearing mice were considered as moribund and euthanized when they developed hind-leg paralysis due to development of paraspinal masses.

### Antibodies and flow cytometry

The following monoclonal anti-mouse antibodies and flow cytometry reagents were obtained from eBioscience (San Diego, CA): anti-CD4 (GK1.5), anti-CD8 (53–6.7), anti-PD-1 (J43), anti-TIM-3 (RMT3–23), anti-LAG-3 (C987W), anti-CD80 (16-10A1), anti-CD44 (1 M7), anti-CD62L (Ly-22), anti-CD127 (A7R34), anti-CD69 (H1.2F3), anti-CD137 (1AH2), anti-OX-40 (OX-86), anti-CD103 (2E7), anti-IFN-γ (XMG1.2), anti-TNF-α (MP6-XT22), anti-Ki-67 (20Raj1), anti-granzyme B (GB11), anti-Foxp3 (FJK-16 s) and propidium iodide staining solution. The following antibodies and reagents were obtained from Biolegend (San Diego, CA): anti-CD8 (53–6.7), anti-PD-1 (J43), anti-TIM-3 (B8.2C12), and anti-CD19 (GD5). Flow cytometric analysis was done on a BD Biosciences LSRII (Franklin Lakes, NJ) flow cytometer, and resulting data analyzed using FlowJo software (Tree Star, Inc.). H-2K^b^/SIINFEKL-PE pentamer and H-2Kb/ SIYRYYGL-PE was purchased from Proimmune, Inc. (Sarasota, FL).

### Interferon-gamma (IFN-γ) ELISPOT assays

To assess frequencies of myeloma-reactive, IFN-γ-secreting CD8^+^ or CD4^+^ T cells, T cells were isolated from spleens and bone marrow by immunomagnetic cell sorting, as previously described [5]. IFN-γ enzyme-linked immunosorbent spot (ELISPOT) assays were done using mouse IFN-γ ELISPOT kits from BD Biosciences, as described earlier [12]. The ELISPOT data was quantified using a Cellular Technology Limited (CTL) ImmunoSpot Analyzer (CTL Analyzers, Cleveland, OH).

### Bio-plex cytokine assays

Flow sorted PD-1^+^ or PD-1^−^ T cells from 5T33 myeloma-bearing mice were activated with plate-bound anti-CD3 mAb (clone 145-2C11, BD Biosciences; 5 μg/mL). Culture supernatants were harvested after 48 h and stored at −80 °C. Thawed supernatants were then analyzed using a murine multiplex cytokine kit (Bio-Rad, Hercules, CA) to detect IL-2, IL-4, IL-5, IL-10, IL-12p70, granulocyte-macrophage colony stimulating factor (GM-CSF), tumor necrosis factor-alpha (TNF-α), and IFN-γ. Cytokines were quantified using a Bio-Plex protein 200 array reader, and data was automatically processed and analyzed using Bio-Plex Manager Software 4.1. Standard curves were generated from recombinant cytokine standards. All samples were assayed in duplicate.

### Intracellular cytokine staining

Intracellular cytokine staining was performed after 6 h of restimulation with 1 μg/ml plate bound anti-CD3 (clone 145-2C11, BD Biosciences) and CD28 (clone 37.51, BD Biosciences) in the presence of GolgiPlug (1 μl/ml; BD Biosciences). Surface staining of cells was performed using a modified FACS buffer containing 10 μg/ml brefeldin A (Sigma-Aldrich). Cells were next stained on ice for 20 min with the primary Abs (anti-CD8, anti-CD4 and anti-CD3), and then intracellularly stained with PE-labeled antibody to IFN-γ, fluorescein isothiocyanate–labeled antibody to granzyme B, or Ki67 and APC-labeled TNF-α. Cells were analyzed by flow cytometry to assess intracellular cytokine expression.

### Statistics

Survival curves were compared using the log-rank (Mantel Cox) test. Data in other experiments were analyzed using the Student’s t test. *P* values ≤0.05 were considered as significant. Statistical analysis was done using Prism version 5.0a software (GraphPad Software, La Jolla, CA).

## Results

### Functional myeloma-reactive cells are present in the PD-1^+^CD8^+^ T cell subset

**Fig. 1:**
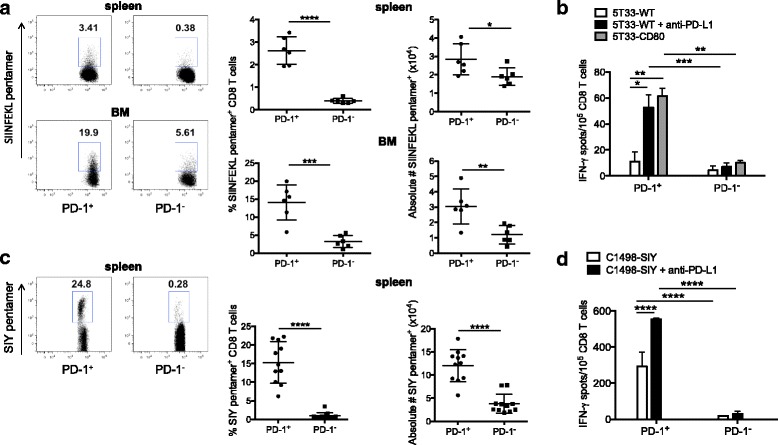
Functional myeloma-reactive cells reside in the PD-1^+^ T cell subset. KaLwRij mice were inoculated with 2 × 10^6^ 5T33-GFP-OVA myeloma cells iv. Mice were euthanized 28 days later, and spleens harvested for analysis**. a** PD-1^+^ and PD-1^−^CD8^+^ T cells were analyzed for SIINFEKL pentamer-positive cells by flow cytometry. The far-left panel depicts a representative example, and the right panels depict percentages and absolute numbers of pentamer-positive CD8 T cells (6 individual mice per group). **b** IFN-γ ELISPOT assay results, where splenic PD-1^+^ and PD-1^−^ CD8^+^ T cells were sorted by flow cytometry and stimulated with wild-type 5T33 (5T33-WT), 5T33-WT plus 10 μg/ml anti-PD-L1 added directly to the assay wells (5T33-WT + anti-PD-L1), or 5T33 cells expressing CD80 (5T33-CD80). The graph is representative of 4 independent experiments**. c** The percentage of SIYRYYGL (SIY) pentamer-positive cells in the spleens of mice bearing C1498-SIY leukemia. The left panel depicts a representative example, and the right panel depicts the results of 11 individual mice per group. **d** IFN-γ ELISPOT results, where splenic PD-1^+^ and PD-1^−^ CD8^+^ T cells were sorted by flow cytometry and stimulated with C1498-SIY myeloma cells or C1498-SIY cells plus 10 μg/ml anti-PD-L1 in the assay wells. The graph is representative of 4 independent experiments. *p ≤ 0.05, **p ≤ 0.01, ***p ≤ 0.001 and ****p ≤ 0.0001

The immunogenic cancer antigens on 5T33 myeloma are unknown. Therefore, to identify T cells with myeloma antigen specificity, we used a 5T33 cell line expressing the model antigen SIINFEKL ovalbumin (OVA) peptide (5T33-GFP-OVA), along with GFP, to facilitate identification of the cells in vivo. To show that PD-1 is up-regulated on myeloma-reactive T cells, KaLwRij mice were inoculated with 2 × 10^6^ 5T33-GFP-OVA cells iv. Mice were euthanized, and spleens and bone marrow harvested 30–35 days after inoculation. CD8^+^ T cells that recognize SIINFEKL were detected by flow cytometry using fluorescently labeled H2K^b^/SIINFEKL pentamers. Our results show that greater percentages and absolute numbers of both spleen and bone marrow PD-1^+^CD8^+^ T cells were SIINFEKL pentamer-positive as compared to PD-1^−^CD8^+^ cells (Fig. 1a). These data directly show that myeloma-specific CD8^+^ T cells are enriched in the PD-1^+^ population.

To examine whether PD-1^+^CD8^+^ T cells secrete cytokine in response to cancer antigen stimulation, IFN-γ ELISPOT assays were performed. For these assays, PD-1^+^CD8^+^ and PD-1^−^CD8^+^ T cells were sorted by flow cytometry and stimulated with 5T33 myeloma cells. While some PD-1^+^CD8^+^ T cells secreted IFN-γ in response to myeloma antigens (Fig. 1b), this number was significantly enhanced either by inclusion of anti-PD-L1 blocking antibody during the assay cell co-culture, or by antigen stimulation with 5T33 myeloma modified to express the co-stimulatory molecule CD80. These data clearly show that the PD-1^+^CD8^+^ T cell subset is enriched in myeloma-reactive T cells, but that many of the cells are relatively inactive in the absence of PD-1 blockade or additional co-stimulation.

To show that PD-1 expression identifies cancer antigen-reactivity in another hematologic malignancy model (C1498 acute myeloid leukemia), the percentages and absolute numbers of cancer-reactive cells were determined, and IFN-γ ELISPOT assays performed on T cells harvested from mice bearing C1498. PD-1^+^CD8^+^ and PD-1^−^CD8^+^ T cells were sorted from the spleens of mice that had been inoculated iv with C1498 cells engineered to express the model peptide antigen peptide SIY (SIYRYYGL; C1498-SIY). As with the 5T33 model, our results show that PD-1^+^CD8^+^ T cells are highly enriched in cancer antigen reactivity (Fig. 1c). Similar to the myeloma model, addition of anti-PD-L1 to the ELISPOT assays resulted in a significant increase in numbers of PD-1^+^CD8^+^ T cells secreting IFN-γ (Fig. 1d).

### PD-1^+^ T cells from myeloma-bearing mice are phenotypically heterogeneous and secrete effector cytokines

In moribund myeloma-bearing (MB) mice, we previously showed that splenic PD-1^+^ T cells stimulated with anti-CD3 exhibit an altered cytokine profile (i.e., secreted less IL-2, IFN-γ and TNF-α) as compared to PD-1^−^ T cells or T cells from non-MB mice [5]. This prompted us to determine if PD-1^+^ T cells co-express markers of T cell dysfunction or activation, or retain the ability to produce effector cytokines when analyzed prior to generation of advanced disease. The phenotype and function of PD-1^+^ T cells was determined 28 days after 5T33 inoculation. This time point is before mice become moribund, which is typically 35–45 days following 5T33 inoculation. At 28 days, myeloma comprises 1–4% of total spleen cells, unlike moribund mice, where approximately 5–20% of the spleen consists of myeloma (data not shown).

**Fig. 2:**
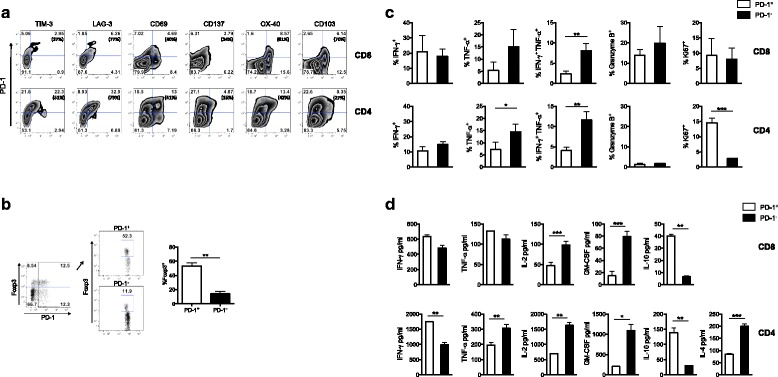
PD-1^+^ T cells from myeloma-bearing mice are phenotypically heterogeneous and secrete effector cytokines. KaLwRij mice were inoculated with 2 × 10^6^ 5T33-GFP cells iv. Spleens were harvested 28 days later for analysis. **a** Flow cytometric analysis of PD-1 co-expression with inhibitory receptors TIM-3 and LAG-3, and activation markers CD69, CD137, OX-40 and CD103 on CD8^+^ and CD4^+^ T cells. **b** Percentages of PD-1^+^Foxp3^+^CD4^+^ T cells analyzed by flow cytometry. **c** T cells were activated with 1 μg/ml plate bound anti-CD3 and anti-CD28 for 6 h, and analyzed for the presence of intracellular cytokines by flow cytometry. **d** Multiplex cytokine analysis of culture supernatants from T cells activated with 5 μg/ml plate bound anti-CD3 (clone 2C11) for 48 h. Data shown are representative of more than four independent analyses. *p ≤ 0.05, **p ≤ 0.01 and ***p ≤ 0.001

The percentage of spleen PD-1^+^CD4^+^ and CD8^+^ T cells in naïve non-myeloma bearing mice is relatively low (~4–7%), as compared to moribund 5T33 bearing mice where 20–60% are PD-1^+^. In naïve mice, only about 1% of PD-1^+^CD8^+^ spleen T cells co-express the checkpoint receptor TIM-3, whereas in moribund 5T33 mice approximately 10% of PD-1^+^CD8^+^ spleen T cells express TIM-3 [5]. For this study, we compared the phenotype of PD-1^+^ and PD-1^−^ T cells from 5T33 bearing mice prior to advanced disease. To characterize PD-1^+^ T cells, spleens were harvested on day 28 and co-expression of PD-1 with various inhibitory and activation molecules was determined by flow cytometry. Figure 2a shows the percentage of total spleen cells co-expressing PD-1 and the other markers tested (upper right quadrant). The bracketed values in each upper right quadrant represent the percentages of PD-1^+^ T cells that co-expressed the marker of interest. Notably, 37% and 77% of PD-1^+^CD8^+^ T cells co-expressed the checkpoint receptors TIM-3 and LAG-3, respectively. However, 81% and 70% of PD-1^+^CD8^+^ T cells also co-expressed activation markers OX40 and CD103, respectively (Fig. 2a, top panel). 34% of CD8^+^PD-1^+^ T cells co-expressed CD137. For PD-1^+^CD4^+^ T cells, 51% and 79% expressed TIM-3 or LAG-3 checkpoint receptors, respectively (Fig. 2a, bottom panel). Of the PD-1^+^CD4^+^ T cells, 52% expressed Foxp3 as compared to approximately 12% of PD-1^−^CD4^−^T cells (Fig. 2b). These data show there are multiple subsets of PD-1^+^CD8^+^ and CD4^+^ T cells expressing both checkpoint receptors and activation markers. T cells that co-express multiple inhibitory receptors have been reported to be dysfunctional relative to cells that express PD-1 alone or no inhibitory receptors [11].

To compare how PD-1^+^ and PD-1^−^ T cells respond functionally to activation signals, cells were sorted into PD-1^+^ and PD-1^−^ T cell subsets and activated with plate-bound anti-CD3 and anti-CD28 for 6 h. This strong activation was used to optimize detection of cytokines produced by the cells. Functional status was assessed by examining presence of IFN-γ, TNF-α, granzyme B and Ki67 by intracellular flow cytometry. For CD8 T cells, there were no statistical differences in percentages of PD-1^+^ T cells expressing intracellular IFN-γ, TNF-α, granzyme B or Ki67 as compared to PD-1^−^ T cells (Fig. 2c, top panel). However, there was a significant decrease in percentages of PD-1^+^CD8^+^ T cells that expressed both IFN-γ and TNF-α as compared to PD-1^−^CD8^+^ T cells. Similar to CD8^+^ T cells, significantly fewer PD-1^+^CD4^+^ T cells co-expressed IFN-γ and TNF-α as compared to PD-1^−^CD4^+^ T cells (Fig. 2c, bottom panel). Significantly lower percentages of PD-1^+^CD4^+^ T cells expressed TNF-α as compared to PD-1^−^CD4^+^ T cells. Surprisingly, the PD-1^+^CD4^+^ T cells had higher Ki67 expression as compared to PD-1^−^CD4^+^ T cells. Overall, these data suggest that in response to strong activation signals, PD-1^+^T cells may be proliferative and they produce similar IFN-γ but less TNF-α as compared to PD-1^−^ T cells.

To further evaluate the ability of PD-1^+^ T cells to produce and secrete effector cytokines, PD-1^+^ and PD-1^−^ T cells were stimulated with plate bound anti-CD3 for 48 h and culture supernatants collected. Supernatants were then analyzed for cytokine content using a multiplex platform. PD-1^−^CD8^+^ and CD4^+^ T cells produced significantly more IL-2 and GM-CSF than PD-1^+^ T cells (Fig. 2d). PD-1^−^CD4^+^ T cells produced significantly more TNF-α than PD-1^+^CD4^+^ T cells. However, the amount of IFN-γ in the PD-1^+^CD8^+^ T cell supernatant was not quantitatively different than that in the supernatant collected from PD-1^−^CD8^+^ T cells. In fact, there was significantly more IFN-γ in the supernatant of PD-1^+^CD4^+^ T cells as compared to PD-1^−^CD4^+^ T cells. Of particular note, both PD-1^+^CD4^+^ and CD8^+^ T cells produced increased amounts of IL-10 as compared to PD-1^−^ T cells. Up-regulation of IL-10 production in IFN-γ-producing PD-1^+^ effector T cells may be a consequence of chronic antigen activation. Co-production of IFN-γ and IL-10 has been reported in Th1 T cells during chronic mouse infections [15, 16].

In summary, prior to advanced 5T33 myeloma burden, there are splenic PD-1^+^ T cells that appeared to be chronically activated, as demonstrated by expression of activation markers CD69, OX-40 and CD103, and inhibitory receptors LAG-3 and TIM-3. When activated, PD-1^+^ T cells expressed the Ki67 proliferation marker, and produced significantly less IL-2, similar or more IFN-γ and more IL-10 than PD-1^−^ T cells.

### PD-1^+^ T cells from myeloma-bearing mice expand in culture and maintain their reactivity

**Fig. 3:**
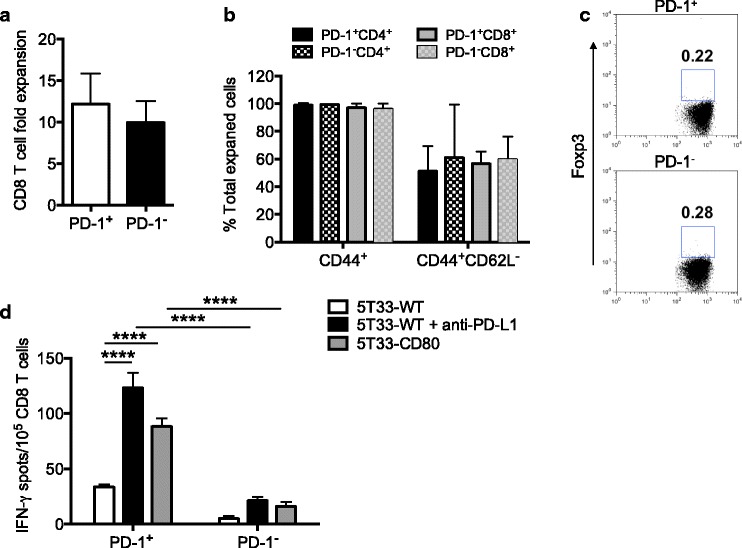
PD-1^+^ T cells from myeloma mice expand ex vivo and secrete IFN-γ in response to myeloma following expansion. **a** Splenic PD-1^+^ and PD-1^−^ CD8^+^ T cells were sorted by flow cytometry, activated with anti-CD3 and anti-CD28, and expanded in culture for 7 days with 20 U/ml IL-2, 5 ng/ml IL-7 and 5 ng/ml IL-15. At the end of expansion, cells were counted and fold-expansion calculated. **b** The percentages of expanded cells expressing CD44 alone or CD44 and low levels of CD62L (CD62L^−^). The graph is representative of 4 independent experiments, 10–12 were mice pooled in each experiment. **c** The percentages of expanded PD-1^+^ or PD-1^−^ CD4^+^ T cells expressing Foxp3. The graph is representative of 4 independent experiments, 5 mice were pooled in each experiment. **d** Frequencies of IFN-γ-producing PD-1^+^ or PD-1^−^ CD8^+^ T cells in response to wild-type 5 T33 myeloma (5T33-WT), 5T33-WT myeloma plus 10 μg/ml anti-PD-L1 (5T33-WT + anti-PD-L1), or 5T33 myeloma cells expressing CD80 (5T33-CD80). The graph is representative of 3 independent experiments. ***p ≤ 0.001

During chronic viral infection and cancer, up-regulation of PD-1 has been shown to be a marker of T cells with reduced ability to proliferate and secrete effector cytokines [17, 18]. In the 5T33 myeloma model we have shown that PD-1^+^ T cells harvested from non-moribund MB bearing mice can be activated to secrete cytokines. However, to use PD-1^+^ T cells for ACT, they must be able to undergo expansion ex vivo and retain effector function. To determine if these qualities persisted in T cells isolated from 5T33-bearing mice, flow cytometric sorted PD-1^+^ and PD-1^−^ CD8 T cells were activated with anti-CD3 and anti-CD28 antibodies and expanded in culture for 7 days with 20 U/ml IL-2, 5 ng/ml IL-7 and 5 ng/ml IL-15. PD-1^+^CD8^+^ T cells expanded in vitro approximately 12-fold after 7 days in culture (Fig. 3a). Almost all expanded cells expressed the CD44 activation marker, and around 50% had a CD44^+^CD62L^−^ effector phenotype (Fig. 3b). Interestingly, PD-1^+^CD4^+^ T cells lost expression of Foxp3 during the expansion (Fig. 3c versus Fig. 2b). To show that expanded T cells maintained effector function, IFN-γ ELISPOT assays were performed. Figure 3d shows that expanded PD-1^+^CD8^+^ T cells secreted IFN-γ in response to myeloma when checkpoint blockade or co-activation through CD80 was provided. The ELISPOT results show that when checkpoint blockade is provided, there are approximately 100 functional myeloma-reactive CD8^+^ T cells for every 10^5^ PD-1^+^CD8^+^T cells. Significantly fewer PD-1^−^CD8^+^ T cells secreted IFN-γ under similar conditions. Together, these data show that within the population of ex vivo expanded PD-1^+^ T cells, around 50% have an activated effector phenotype, few of the cells are CD4^+^Foxp3^+^_,_ and 5T33-reactive PD-1^+^CD8^+^ T cells secrete IFN-γ.

### ACT with cultured PD-1^+^ CD8^+^ and CD4^+^ T cells eliminates myeloma in vivo

**Fig. 4:**
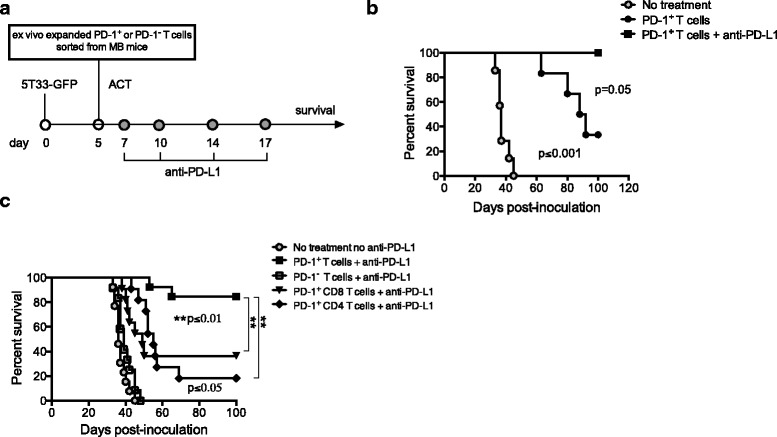
PD-1^+^ T cells expand ex vivo and provide anti-myeloma immunity when given as ACT. **a** Experimental design. At day 0, Rag-1-deficient recipient mice were inoculated with 10^6^ 5T33-GFP cells iv. Five days later, mice received ex vivo-expanded T cells as ACT. Some mice also received 125 μg anti-PD-L1 intraperitoneally (ip) at the indicated time points. Control mice received no treatment. **b** Survival curves of mice treated with ACT consisting of 3-4 × 10^6^ PD-1^+^ T cells at a CD4:CD8 ratio of 1:1 with or without 125 μg anti-PD-L1. Moribund mice were euthanized**.** Data are combined from 2 independent experiments, with *n* = 6–7 mice per experimental group. **c** Survival curves of mice given the following: (1) No treatment, (2) 3-4 × 10^6^ PD-1^+^ CD4^+^ and CD8^+^ T cells at a ratio of 1:1 [PD-1^+^ T cells group], (3) 3-4 × 10^6^ PD-1^−^ CD4^+^ and CD8^+^ T cells at a ratio of 1:1 [PD-1^−^ T cells group], (4) 1.5-2 × 10^6^ PD-1^+^ CD8^+^ T cells alone, or (5) 1.5-2 × 10^6^ PD-1^+^ CD4^+^ T cells alone. All mice, except the ‘no treatment’ group, received 125 μg anti-PD-L1 ip on days 7, 10, 14 and 17 after myeloma inoculation. The data are combined from 3 to 4 independent experiments, with *n* = 11–15 mice per experimental group.

To examine whether PD-1^+^ T cells could provide anti-myeloma immunity in vivo, cultured/expanded cells were infused into MB C57BL/6-Rag-1-deficient mice as ACT. Rag-1-deficient mice were chosen for these experiments to avoid the need for preconditioning (i.e., WBI), and to permit analysis of individual T cell subsets that were infused as ACT. Rag-1-deficient mice were inoculated with 10^6^ 5T33-GFP myeloma cells iv. Five days later, mice were given ACT with 3-4 × 10^6^ PD-1^+^CD4^+^ and CD8^+^ T cells at a CD4:CD8 ratio of 1:1. Since our IFN-γ ELISPOT data demonstrated that myeloma-reactive PD-1^+^ T cells required PD-L1 blockade to enhance IFN-γ secretion, some mice also received anti-PD-L1 antibody intraperitoneally on days 7, 10, 14 and 17 (Fig. 4a). Mice were then followed for survival and euthanized when moribund. Mice given no treatment died within 40 days after 5T33 inoculation (Fig. 4b). There was a significant delay in cancer progression in mice that received ACT of expanded PD-1^+^ T cells, and about 30% of these mice survived beyond 100 days. Co-administration of expanded PD-1^+^ T cells and anti-PD-L1 further improved survival and eliminated myeloma in 100% of mice (Fig. 4b), demonstrating that ongoing PD-L1 blockade was needed to achieve optimal efficacy.

**Fig. 5:**
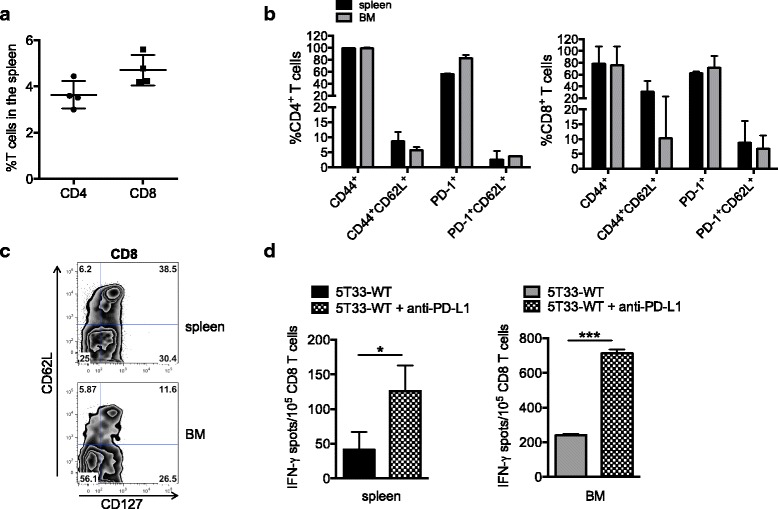
Adoptively transferred PD-1^+^ T cells persist in vivo and retain effector function. From the experiments in Fig. 4, four mice that received PD-1^+^ T cells and eliminated myeloma received a re-challenge of 2 × 10^6^ 5T33 myeloma cells 120 days after the initial myeloma inoculation. Five days later, spleens and bone marrow were harvested for analysis. **a** The percentages of CD4^+^ and CD8^+^ T cells detected in spleens by flow cytometry. **b** The percentages of CD4^+^ and CD8^+^ T cells harvested from the spleen and bone marrow expressing the indicated activation markers, memory markers, and PD-1. **c** Flow cytometric histograms showing expression of the memory marker CD127 on CD8^+^ T cells harvested from spleen and BM. Data represents pooled T cells from one experiment. **d** CD8^+^ T cells isolated from spleen or bone marrow (BM) by immunomagnetic sorting were tested in IFN-γ ELISPOT assays upon stimulation with wild-type 5T33 myeloma (5T33-WT) or 5T33-WT plus 10 μg/ml anti-PD-L1 (added to the assay wells). The graph depicts representative results from 2 independent experiments.

Next, we compared the anti-myeloma efficacy of different cultured/expanded T cell subsets given as ACT. Since PD-L1 blockade synergized with ACT to produce more effective cancer regression in Fig. 4b, all mice given ACT were treated with anti-PD-L1 for this study. Rag-deficient mice were treated as in Fig. 5a. Mice received the following T cell subsets: (1) combined 1:1 ratio of PD-1^+^ CD4^+^ and CD8^+^ T cells, (2) combined 1:1 ratio of PD-1^−^ CD4^+^ and CD8^+^ cells, (3) PD-1^+^CD8^+^ T cells alone, or (4) PD-1^+^CD4^+^ T cells alone. For condition #3 (PD-1^+^CD8^+^ T cells alone), we were able to calculate from the ELISPOT data in Fig. 3d that there were approximately 20,000 functional myeloma-specific PD-1^+^CD8^+^ T cells infused. As observed in the previous experiment, mice that did not receive ACT died within 50 days after myeloma inoculation. Ninety percent of mice given the combination of PD-1^+^CD4^+^ and CD8^+^ T cells survived for 100 days (Fig. 4c). In contrast, none of the mice treated with PD-1^−^CD4^+^ and CD8^+^ T cells survived past day 50 after myeloma inoculation (Fig. 4c). These data provide compelling evidence that PD-1^+^ T cells provide anti-myeloma reactivity in vivo. Furthermore, while the PD-1^+^CD4^+^ and CD8^+^ T cell subsets each contained anti-myeloma reactivity, the combination of PD-1^+^CD4^+^ and CD8^+^ T cells provided the best anti-myeloma effect.

### Adoptively transferred PD-1^+^ T cells persist in recipient mice and provide a long-term anti-myeloma response

The in vivo anti-myeloma immunity provided by the adoptively transferred PD-1^+^ T cells prompted us to test whether the cells persisted and were capable of providing memory. To test this, mice given PD-1^+^ T cells as ACT that had eliminated established 5T33 myeloma were re-challenged with 2 × 10^6^ 5T33 myeloma cells 120 days after the initial inoculation. Five days after myeloma re-challenge, spleens and bone marrow were harvested to analyze persisting T cells. Figure 5a shows the percentages of CD8^+^ (4.7%) and CD4^+^ (3.6%) T cells detected in the spleens by flow cytometry. Phenotypic analysis of surviving CD8^+^ T cells harvested from both the spleen and bone marrow is shown in Fig. 5b. Most of the transferred cells remained activated as indicated by CD44 expression (Fig. 5b). Importantly, both CD4^+^ and CD8^+^ T cells with a memory phenotype (CD44^+^CD62L^+^) were present in both the spleen and bone marrow. PD-1 was expressed on greater than 50% of splenic and 75% of bone marrow CD8^+^ T cells. The memory marker CD127 (IL-7Rα) was assessed on one cohort of pooled mice. Figure 5c shows expression of CD127 on CD8^+^ T cells harvested from both the spleen and bone marrow. IFN-γ ELISPOT assays were also performed on both spleen and bone marrow-derived T cells to assess anti-myeloma function. CD8^+^ T cells were isolated by immunomagnetic cell sorting and stimulated with wild-type 5T33 myeloma (5T33-WT) or 5T33-WT plus 10 μg/ml anti-PD-L1 in the assay wells (5T33-WT + anti-PD-L1). T cells from spleen and bone marrow produced IFN-γ in response to myeloma (Fig. 5d). As shown previously, IFN-γ production was increased when anti-PD-L1 was added to the assay wells. These data show that when PD-1^+^CD4^+^ and CD8^+^ T cells are adoptively transferred into Rag1-deficient mice, they remain activated long term with some cells expressing memory markers.

## Discussion

ACT holds promise as an anti-cancer immune therapy targeting malignancies with heterogeneous mutational landscapes, but it must be optimized to induce more effective anti-cancer responses. The potency of ACT is dependent on the infusion of T cells with cancer antigen specificity as well as the ability to reverse functional impairment (i.e., exhaustion) acquired by chronically activated T cells [19]. In this study, we confirmed that cancer antigen-specific CD8^+^ T cells are enriched in the PD-1^+^ subset in the setting of murine hematologic malignancies (Fig. 1). When activated with polyclonal stimulation, PD-1^+^ T cells produced IFN-γ similar to PD-1^−^ T cells, however the PD-1^+^ T cells had a unique cytokine profile secreting both IFN-γ and IL-10. In vivo, anti-myeloma immunity was conferred by ACT with PD-1^+^ T cells, but only when combined with PD-1 checkpoint blockade (Figs. 4 and 5). Together, these data show that PD-1^+^ T cells are cancer-reactive, can be expanded ex vivo, secrete Th1 cytokines, and are functional in vivo. The unique cytokine profile, the in vitro increase in IFN-γ production in the presence of checkpoint blockade, as well as the requirement of checkpoint blockade for in vivo anti-myeloma immunity, suggest that PD-1^+^ T cells are functionally impaired, but the dysfunctional state can be reversed to provide anti-myeloma immunity [20].

Certain markers have been associated with dysfunctional or exhausted T cells (T_ex_). Recently, CD8^+^ T_ex_ cells have been characterized in human melanoma. These cells express multiple markers such as Ki67^+^, Eomes^hi^, Tbet^lo^, CD39^+^, CD27^+^, CD45RA^lo^ and multiple checkpoint receptors (PD-1, TIM-3, LAG-3, 2B4) [21, 22]. In melanoma patients, treatment with anti-PD-1 (pembrolizumab) reversed the T_ex_ phenotype. In a chronic viral murine model, CD8 T cells that were CXCR5^+^Tcf1^+^ TIM-3^−^ were not terminally exhausted, but rather acted as stem cells during chronic infection [23]. It would be interesting to know if PD-1^+^ T cells express these markers. In our study, we show in MB mice the presence of multiple PD-1^+^CD4^+^ and CD8^+^ T cell subsets present in spleen (Fig. 2a). Interestingly, on both CD4^+^ and CD8^+^ T cells, PD-1 was co-expressed with other checkpoint receptors (TIM-3 and LAG-3), but there were also cells that co-expressed PD-1 with activation markers (CD69, OX-40 and CD103). Given the multiplicity of PD-1^+^ T cell subsets, identifying the phenotype of PD-1^+^ T cells that are T_ex_ will require an in depth phenotypic analysis. Whether there are subsets of effector PD-1^+^ T cells with the ability to proliferate in vivo and provide in vivo anti-myeloma immunity, or whether PD-1^+^ T_ex_ cells revert to effector cells (T_eff_) in the presence of strong activation signals, are questions yet to be answered.

In the current study, spleen PD-1^+^CD8^+^ T cells activated with anti-CD3 produced IFN-γ comparable to PD-1^−^CD8^+^ T cells (Fig. 2b and c). These data contradict previous data shown by Hallett et al., where IFN-γ was not produced by anti-CD3 activated PD-1^+^CD8^+^ T cells harvested from 5T33 ‘moribund’ mice [5]. These data suggest that as myeloma burden progresses to a moribund state, the ability of PD-1^+^CD8^+^ T cells to secrete IFN-γ decreases even in the presence of strong T cell receptor activation. Despite the production of Th1 cytokines when exposed to strong activating signals (i.e., anti-CD3 or anti-CD3 plus anti-CD28), the cytokine profile of PD-1^+^ T cells differed from their PD-1^−^ counterparts (Fig. 2c and d). Most notable, both CD4^+^ and CD8^+^ PD-1^+^ T cells secreted IL-10 in addition to IFN-γ. CD4^+^ T cells that secrete both IFN-γ and IL-10 have been previously described. In a mouse model of systemic *T. gondii* infection, IL-10-producing CD4^+^ T cells were characterized as effector cells that simultaneously produced IFN-γ [16]. These cells displayed potent effector function against *T. gondii,* but also suppressed production of IL-12 by antigen-presenting cells. Interestingly, IL-10 expression was induced in Th1 CD4^+^ T cells after recent antigen exposure. The observation that myeloma-reactive PD-1^+^ CD4^+^ and CD8^+^ T cells secrete both IFN-γ and IL-10 suggests these cells may be at the crossroads of an immune switch from effector to tolerogenic [24]. The regulation and role of IL-10 produced from myeloma-reactive PD-1^+^ T cells is entirely unknown. Unraveling the mechanistic impact of IL-10 production in myeloma-reactive or cancer-reactive effector T cells has major relevance for optimizing immunotherapy.

For in vivo studies, we used Rag1-deficient mice as recipients of PD-1^+^ T cell adoptive therapy to assess anti-myeloma efficacy. This model system was ideal as it provided a lymphopenic setting without confounding effects from endogenous T cells. We have previously shown lymphopenia is a requirement for the activation of myeloma-specific T cells or effective ACT with myeloma antigen-experienced T cells [5, 6]. There are multiple mechanisms by which endogenous T cells could interfere with the anti-myeloma effect provided by PD-1^+^ T cells. These include consumption or production of cytokines, activation into effectors, and the presence of T regulatory cells. Following ACT, adoptively transferred PD-1^+^ T cells persisted in vivo over 100 days (Fig. 5). Transferred cells remained activated and functional with small percentages of CD44^+^CD62L^+^ putative memory cells present.

## Conclusions

In summary, we show that PD-1^+^ T cells harvested from MB mice contain the vast majority of cancer antigen-reactive T cells. Furthermore, these cells can be ex vivo expanded to serve as functional effector cells when given as ACT in the context of lymphopenia and checkpoint blockade. These observations advance the field in two ways. First, this data provides evidence that PD-1 can be used serve as a marker for both cancer antigen reactive CD8 and CD4 T cells in hematologic malignancies. Second, these results clearly show that PD-1^+^ cancer antigen-reactive T cells can be used for effective ACT in vivo, but that continuous blockade of the PD-1 pathway is necessary for optimal efficacy.

## Abbreviations

ACT: Adoptive cell therapy
CIITA: Class II transactivator
MB: Myeloma-bearing
OVA: Ovalbumin
PD-1: Programmed death receptor-1
PD-L1: Programmed death receptor ligand-1
SIY: SIYRYYGL
TILs: Tumor-infiltration lymphocytes
WBI: Whole body irradiation

## References

[1] Iwai Y, Hamanishi J, Chamoto K, Honjo T (2017). Cancer immunotherapies targeting the PD-1 signaling pathway. J Biomed Sci.

[2] Ansell SM, Lesokhin AM, Borrello I, Halwani A, Scott EC, Gutierrez M (2015). PD-1 blockade with nivolumab in relapsed or refractory Hodgkin's lymphoma. N Engl J Med.

[3] Xia Y, Medeiros LJ, Young KH (2016). Immune checkpoint blockade: releasing the brake towards hematological malignancies. Blood Rev.

[4] Topalian SL, Drake CG, Pardoll DM (2015). Immune checkpoint blockade: a common denominator approach to cancer therapy. Cancer Cell.

[5] Hallett WH, Jing W, Drobyski WR, Johnson BD (2011). Immunosuppressive effects of multiple myeloma are overcome by PD-L1 blockade. Biol Blood Marrow Transplant.

[6] Kearl TJ, Jing W, Gershan JA, Johnson BD (2013). Programmed death receptor-1/programmed death receptor ligand-1 blockade after transient lymphodepletion to treat myeloma. J Immunol.

[7] Baitsch L, Baumgaertner P, Devêvre E, Raghav SK, Legat A, Barba L (2011). Exhaustion of tumor-specific CD8^+^ T cells in metastases from melanoma patients. J Clin Invest.

[8] Day CL, Kaufmann DE, Kiepiela P, Brown JA, Moodley ES, Reddy S (2006). PD-1 expression on HIV-specific T cells is associated with T-cell exhaustion and disease progression. Nature.

[9] Gros A, Robbins PF, Yao X, Li YF, Turcotte S, Tran E (2014). PD-1 identifies the patient-specific CD8(+) tumor-reactive repertoire infiltrating human tumors. J Clin Invest.

[10] Andersen R, Donia M, Ellebaek E, Borch TH, Kongsted P, Iversen TZ (2016). Long-lasting complete responses in patients with metastatic melanoma after adoptive cell therapy with tumor-infiltrating lymphocytes and an attenuated IL2 regimen. Clin Cancer Res.

[11] Blackburn SD, Shin H, Haining WN, Zou T, Workman CJ, Polley A (2009). Coregulation of CD8+ T cell exhaustion by multiple inhibitory receptors during chronic viral infection. Nat Immunol.

[12] Jing W, Gershan JA, Weber J, Tlomak D, McOlash L, Sabatos-Peyton C (2015). Combined immune checkpoint protein blockade and low dose whole body irradiation as immunotherapy for myeloma. J Immunother Cancer.

[13] Gros A, Parkhurst MR, Tran E, Pasetto A, Robbins PF, Ilyas S (2016). Prospective identification of neoantigen-specific lymphocytes in the peripheral blood of melanoma patients. Nat Med.

[14] Inozume T, Hanada K-I, Wang QJ, Ahmadzadeh M, Wunderlich JR, Rosenberg SA (2010). Selection of CD8+PD-1+ lymphocytes in fresh human melanomas enriches for tumor-reactive T cells. J Immunother.

[15] Anderson CF, Oukka M, Kuchroo VJ, Sacks D (2007). CD4(+)CD25(−)Foxp3(−) Th1 cells are the source of IL-10-mediated immune suppression in chronic cutaneous leishmaniasis. J Exp Med.

[16] Jankovic D, Kullberg MC, Feng CG, Goldszmid RS, Collazo CM, Wilson M (2007). Conventional T-bet(+)Foxp3(−) Th1 cells are the major source of host-protective regulatory IL-10 during intracellular protozoan infection. J Exp Med.

[17] Kao C, Oestreich KJ, Paley MA, Crawford A, Angelosanto JM, Ali MA (2011). Transcription factor T-bet represses expression of the inhibitory receptor PD-1 and sustains virus-specific CD8+ T cell responses during chronic infection. Nat Immunol.

[18] Virgin HW, Wherry EJ, Ahmed R (2009). Redefining chronic viral infection. Cell.

[19] Pauken KE, Wherry EJ (2015). Overcoming T cell exhaustion in infection and cancer. Trends Immunol.

[20] Barber DL, Wherry EJ, Masopust D, Zhu B, Allison JP, Sharpe AH (2006). Restoring function in exhausted CD8 T cells during chronic viral infection. Nature.

[21] Gupta PK, Godec J, Wolski D, Adland E, Yates K, Pauken KE (2015). CD39 expression identifies terminally exhausted CD8+ T cells. PLoS Pathog.

[22] Huang AC, Postow MA, Orlowski RJ, Mick R, Bengsch B, Manne S (2017). T-cell invigoration to tumour burden ratio associated with anti-PD-1 response. Nature.

[23] Im SJ, Hashimoto M, Gerner MY, Lee J, Kissick HT, Burger MC (2016). Defining CD8+ T cells that provide the proliferative burst after PD-1 therapy. Nature.

[24] Mingomataj EC, Bakiri AH (2016). Regulator versus Effector paradigm: interleukin-10 as indicator of the switching response. Clin Rev Allergy Immunol.

